# A Theoretical Study on the Underlying Factors of the Difference in Performance of Organic Solar Cells Based on ITIC and Its Isomers

**DOI:** 10.3390/molecules28196968

**Published:** 2023-10-07

**Authors:** Si-Qi Huang, Li-Li Wang, Qing-Qing Pan, Zhi-Wen Zhao, Ying Gao, Zhong-Min Su

**Affiliations:** 1 College of Chemical Engineering, Hubei University of Arts and Science, Xiangyang 441053, China; huangsiqi61@outlook.com; 2 Jilin Provincial Key Laboratory of Straw–Based Functional Materials, Institute for Interdisciplinary Biomass Functional Materials Studies, Jilin Engineering Normal University, Changchun 130052, China; 18523778447@163.com; 3 School of Chemistry and Environmental Engineering, Changchun University of Science and Technology, Jilin Provincial Science and Technology Innovation Center of Optical Materials and Chemistry, Jilin Provincial International Joint Research Center of Photo-Functional Materials and Chemistry, Changchun 130022, China; panqq349@nenu.edu.cn (Q.-Q.P.); zmsu@nenu.edu.cn (Z.-M.S.); 4 State Key Laboratory of Supramolecular Structure and Materials, Institute of Theoretical Chemistry, College of Chemistry, Jilin University, Changchun 130021, China

**Keywords:** OSC, non-fullerene acceptor, acceptor, DFT, MD

## Abstract

Recently, non-fullerene-based organic solar cells (OSCs) have made great breakthroughs, and small structural differences can have dramatic impacts on the power conversion efficiency (PCE). We take **ITIC** and its isomers as examples to study their effects on the performance of OSCs. **ITIC** and **NFBDT** only differed in the side chain position, and they were used as models with the same donor molecule, **PBDB-T**, to investigate the main reasons for the difference in their performance in terms of theoretical methods. In this work, a detailed comparative analysis of the electronic structure, absorption spectra, open circuit voltage and interfacial parameters of the **ITIC** and **NFBDT** systems was performed mainly by combining the density functional theory/time-dependent density functional theory and molecular dynamics simulations. The results showed that the lowest excited state of the **ITIC** molecule possessed a larger ∆*q* and more hybrid FE/CT states, and **PBDB-T/ITIC** had more charge separation paths as well as a larger *k*_CS_ and smaller *k*_CR_. The reason for the performance difference between **PBDB-T/ITIC** and **PBDB-T/NFBDT** was elucidated, suggesting that **ITIC** is a superior acceptor based on a slight modulation of the side chain and providing a guiding direction for the design of superior-performing small molecule acceptor materials.

## 1. Introduction

**Figure 1:**
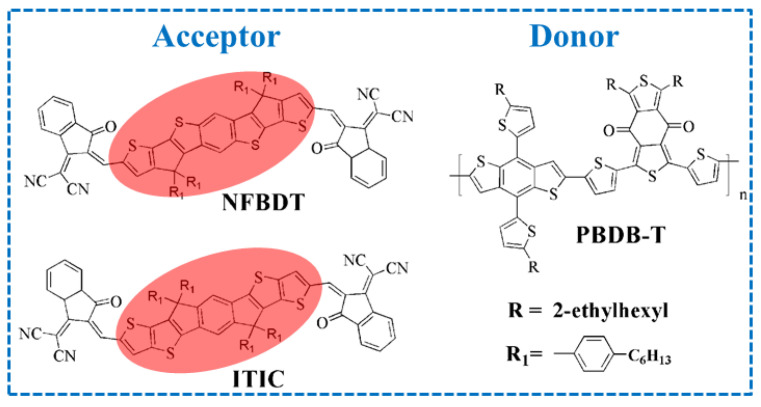
Chemical structure of donor **PBDB-T**, acceptor **NFBDT** and its isomer **ITIC**, with different donor units marked red.

As a promising renewable photovoltaic technology, organic solar cells (OSCs) can directly convert sunlight and electricity, with significant advantages such as a low cost, light weight, flexibility, adjustable optical transparency, etc. [1,2,3]. As the best of OSC device architectures so far, the bulk heterojunction (BHJ), which is vital to achieve efficient charge extraction and transport, is composed with a blend of a donor and an acceptor [4]. Due to the rapid development of small molecule acceptors, OSCs have achieved high power conversion efficiencies (PCEs) of ≈19%[5]. Totally, acceptors are classified into two categories, which are fullerene acceptors (FAs) and non-fullerene acceptors (NFAs). Fullerene has been an efficient acceptor in past decades, but its inherent properties including weak light absorption and limited chemical structural tunability have posed limitations on its further development. Meanwhile, the high cost and poor thermal stability of FA-based OSCs are not conducive to the commercialization progress of OSCs [6]. Compared with FAs, NFAs with stronger and wider absorption, precisely tuned bandgap, crystallization properties and ease of synthesis are regarded as superior materials [7]. Recently, NFAs, which contain the A-D-A and A-DA’D-A structures, have been utilized widely and play a pivotal role in advancing OSC performance [8,9]. For instance, perylene diimide (**PDI**) dimers of **bis-PDI-T-EG** linked through thiophene reduced aggregation and resulted in a PCE of 4.03% with the donor of **PBDTTT-CT** relative to the **PDI** monomer [10]. The **TPH** NFA and selenium-annulated **TPH-Se** with fused **PDI** monomers were synthesized. In particular, its single-crystal architectures exhibited a propeller-like 3D network that accelerates electron transport, with the PCEs reaching 8.28% and 9.28%, respectively [11]. In 2015, Zhan et al. reported a novel A-D-A 3,9-bis(2-methylene-(3-(1,1-dicyanomethylene)-indanone))-5,5,11, 11-tetrakis (4-hexylphenyl)-dithieno [2,3-d:2′,3′-d′]-s-indaceno [1,2-b:5,6-b′]dithiophene (**ITIC)** featuring a dithieno [2,3d:2′,3′d′]-sindaceno [1,2b:5,6b′]dithiophene (**IDTT**) core and an end group of 2-methylene-(3-(1,1-dicyanomethylene) indanone) (**IC**) [12]. However, based on the limited spectral overlap in absorption, **PTB7-Th:ITIC** exhibited a PCE of 6.8%. Subsequently, **ITIC** was blended with the wide-bandgap **J71**, and the **PBDB-T** polymer achieved PCEs of 11.41% and 11.21%, respectively [13,14]. In order to enhance the efficiency of OSCs, introducing the electron-donating or electron-withdrawing units in the terminal groups, which can tune the energy level and morphology, were proposed as promising strategies. Hou et al. synthesized **IT-M** and **IT-OM2** with methyl and methoxyl units substituted on the **IC** terminal group, resulting in an upward shift in the LUMO/HOMO levels. The PCEs of **PBDB-T:IT-M** and **PBDB-T:IT-OM2** reached 12.5% and 11.9%, respectively [15,16]. Furthermore, a four-fluorine atom was introduced to the **IC** group (named as **IT-4F**), enhancing the charge transport and intermolecular interactions. A high PCE of 14.7% was achieved based on **PTO2:IT-4F**, and a higher PCE (15.3%) with another donor, **PFBCPZ** for **IT-4F**-based OSCs, was reported [17,18]. Chen and co-workers reported **NFBDT** with a heptacyclic benzodi(cyclopentadithiophene) (**FBDT**) core based on **BDT**, with 2-(2,3-dihydro-3-oxo-1H-inden-1-ylidene) propanedinitrile (**INCN**) as the terminal group. **NFBDT** not only has a planar backbone, but also reasonable aggregation at the solid state, and it is an isomer of **ITIC** with the differences of the side chain position in the donor unit (shown in Figure 1). However, **PBDB-T:NFBDT** devices showed a PCE of 8.80%, which is lower than **PBDB-T:ITIC** OSCs (11.21%) [14,19]. Based on extensive experimental data, small differences in the donor unit generate large differences in the PCE, but the intrinsic influences remain ambiguous [20,21,22,23]. Actually, the donor/acceptor (D/A) interface has dominated the charge separation efficiency, which is related with the PCE in OSCs [24].

In this work, molecular dynamics simulations (MD) and the density functional theory/time-dependent density functional theory (DFT/TDDFT) methods were combined to probe factors influencing the performance differences for **PBDB-T:ITIC** and **PBDB-T:NFBDT** systems. The geometry optimization, absorption spectra, open-circuit voltage (*V*_OC_) and important parameters at the interface were analyzed. The results could provide theoretical guidance for designing efficient acceptor materials.

## 2. Computational Methods

### 2.1. MD Simulations

MD simulations were utilized to simulate the **PBDB-T/ITIC** and **PBDB-T/NFBDT** BHJ interfaces with general AMBER force field (GAFF) in Gromacs software package [25]. The GAFF with the restricted electrostatic potential (RESP) [26,27] charge was established for all molecules at Hartree–Fock/6-31G (d, p). According to the experiment [19], the best D/A weight ratio was 1:0.8 for two systems. The simulation systems were subjected to initial energy minimization, followed by a 3 ns canonical (NVT) ensemble, incorporating long-range electrostatics using Particle Mesh Ewald (PME) and van der Waals interactions with a cutoff of 0.1 Å. Whole MD simulations were conducted throughout the leap-frog integrator with a time step of 1 fs at 300 k and 1 bar. Additionally, Nosé–Hoover thermostat [28,29] and Parrinello–Rahman barostat [30] were used to control temperature and pressure, respectively. When an equilibration was reached in NVT, the isothermal–isobaric (NPT) ensemble at 300 k and 1 bar for 10 ns were subsequently adopted to simulate interfacial morphologies until the system reached equilibrium. From all potential curves, the **PBDB-T/ITIC** and **PBDB-T/NFBDT** reached equilibrium states with minimum energy via NVT and NPT ensemble simulation (Figures S1 and S2). Therefore, the initial geometry structures of D/A interface models with good π-π stacking were extracted from the final cluster models after MD simulation (shown Figure S3), respectively. The selection of these clusters was performed using the quantum mechanical/molecular mechanics (QM/MM) method [31], taking into account the influence of the surrounding environment. The QM part was treated at the B3LYP/6-31G (d, p) level.

### 2.2. Quantum Chemical Calculations

The optimizations and frontier molecular orbital (FMO) energy levels of **ITIC** and **NFBDT** were computed using the B3LYP/6-31G (d, p) [32], which can provide reliable electronic structures for organic small molecules [33,34]. A solvent (chloroform) effect was considered using a polarizable continuum model (PCM) during TD-DFT calculations. The absorption properties were calculated using PBE0/6-31G(d, p) level based on the ground-state geometries, which was consistent with experimental values for **ITIC** and **NFBDT** (Figure S4). Furthermore, the CAM-B3LYP function presented good description for estimating the excitation energies in small compounds, and all excited state calculations were evaluated at the CAM-B3LYP/6-31G(d, p) level in the TD-DFT theory [35]. The charge transfer properties were computed using the semi-classical Marcus theory [36]:

$$k=\left(\frac{{4\pi }^{2}}{h}\right)V_{DA}^{2}\left(\frac{1}{\sqrt{4\pi \lambda k_{B}T}}\right)exp\left[\frac{{-\left(∆G+\lambda \right)}^{2}}{4\lambda k_{B}T}\right]$$

where *V_DA_* is the transfer integral between the initial and last states, ∆*G* denotes the Gibbs free energy difference, *λ* denotes the reorganization energy, T denotes the temperature (generally set as 300 K), and *k_B_* and *h* denote the Boltzmann and Plank constants, respectively. Here, the reorganization energy (*λ*), which comprises an internal component from intramolecular vibrations *λ*_int_ and an external part affected by the surrounding medium *λ*_s_, was evaluated using the method from references [37,38,39,40]. In addition, the electronic coupling in the charge separation (CS) process and charge recombination (CR) process [40] were computed via CAM-B3LYP/6-31G(d, p) in the Q-Chem 4.0 software [41] with the Generalized Mulliken–Hush (GMH) method [42]. The absorption spectra and the charge density difference (CDD) maps were visualized via Multiwfn 3.8 code [43]. Additionally, the quantum chemical calculations were performed using the Gaussian 09 program package [44].

## 3. Results and Discussion

### 3.1. Properties Related to Ground State

**Figure 2:**
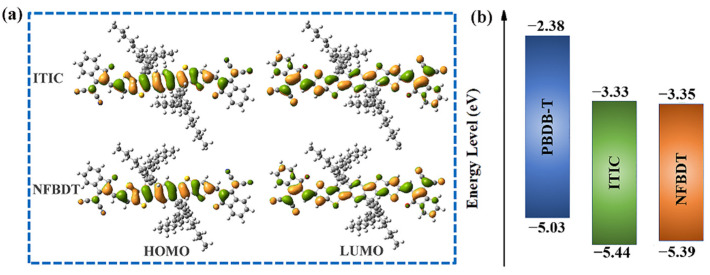
(**a**) Frontier molecular orbital (FMO) distributions of **ITIC** and **NFBDT** molecules. The green color represents the positive and negative phase, respectively. (**b**) Energy levels of donors and acceptors.

The geometric structures have important impacts on the photoelectric properties. As shown in Figure S5, the optimized side and half side of **ITIC** and **NFBDT** show that the bulk of **ITIC** has good planarity, which is consistent with the results of the experiment. The position of the side chain of **NFBDT** is closer to the end groups, and the steric hindrance makes the end group twist at a small angle with the donor unit. However, the skeletons of isomers are quite planar with the large steric hindrance of side chains, which is favorable to increase end-group π-π stacking, especially for **ITIC**. The π-π stacking of the electron-withdrawing end groups in A-D-A acceptors can considerably increase the energy splitting of the singlet state and further obtain a reduction in the ∆*E*_ST_, which is effective to suppress the triplet recombination channel, finally leading to a high FF for OSCs [45]. Recently, it was proposed that the A-D-A molecules will pre-aggregate via end-group π–π stacking, promoting a greater tendency for molecules to form the horizontal and face-on orientations [46]. Furthermore, both the highest occupied molecular orbital (HOMO) and lowest unoccupied molecular orbital (LUMO) are delocalized (Figure 2). Relative to the HOMO orbital, the LUMO orbital is distributed on the terminal benzene rings. Obviously, the electronic structure of the two molecules is similar.

The energy driving force (Δ*E*) is provided from the energy offsets of the HOMO level and LUMO level between the donors and acceptors, which is essential to provide excess energy to the charge transfer (CT) state for effective CS. The Δ*E* > 0.3 eV at the D/A interface will greatly increase the probability of free carriers formed via exciton dissociation [45]. Herein, the computed values of the HOMO and LUMO for **PBDB-T**, **ITIC** and **NFBDT** are −5.03, −5.44 and −5.39 eV and −2.38, −3.33 and −3.35 eV, respectively. Δ*E*_LUMO_ and Δ*E*_HOMO_ of the **PBDB-T/ITIC** and **PBDB-T/NFBDT** systems are 0.95 and 0.97 eV and 0.41 and 0.36 eV (Table S1), respectively, all exceeding 0.3 eV; thus, two systems have sufficient Δ*E* to achieve efficient exciton dissociation at the D/A interface. In addition, the HOMO and the LUMO are lower lying than **PBDB-T**; thus, they can receive electrons from **PBDB-T** or deliver holes to **PBDB-T**. Meanwhile, the HOMO level of **ITIC** is lower than **NFBDT**, which is favorable for hole transport. The reason for the difference in the HOMO and LUMO of **NFBDT** and **ITIC** is mainly the differences in the side chain positions of both acceptors.

*V*_OC_ is one of the important parameters used to measure the PCE of OSCs, which is closely related to the energy level arrangement of the donor and acceptor. Obviously, the lower the HOMO energy level of the donor, the higher the LUMO energy level of the acceptor, and the higher the *V*_OC_ of the OSCs. Due to the slightly higher LUMO energy level, **ITIC** has a slightly larger *V*_OC_ (Table S1). However, the low LUMO levels of the NFAs are conducive to the air-stable electron transmission and the avoidance of electro-chemical oxidation reactions with **H_2_O** and **O_2_**, which helps to improve the stability of OSCs [47].

### 3.2. Properties Related to Excited State

The amount of transfer charge (∆*q*) for the excited states are calculated via Multiwfn 3.8. The excited states in the OSCs can be classified into three categories through the value of ∆*q*: (1) the Frankel exciton (FE) state, which has a ∆*q* value between 0 and 0.3 |e|; (2) the CT state, whose ∆*q* exceeds 0.7 |e|; and (3) the hybrid charge transfer (HCT) state, namely the mixture of two formers, whose ∆*q* is distributed from 0.3 to 0.7 |e| [47]. In all excited states, the lowest singlet excited state (S_1_) corresponds to the FE state with the largest oscillation intensity and makes an important contribution to the CS process, and it is calculated as the FE state in this system. Here, only the S_0_→S_1_ of the acceptors are calculated, and the ∆*q* values of the acceptors are 0.646 and 0.615 |e|, which indicates that S_1_ exhibits HCT properties and facilitates the charge separation. Compared with **NFBDT**, **ITIC** has a larger ∆*q*, indicating that it may have a higher charge separation efficiency.

The degree of overlap between the absorption spectra and the solar absorption range is closely related to the short-circuit current (*J*_SC_) of OSCs. As shown in Figure S4, **ITIC** and **NFBDT** have two absorption peaks, 423.3 and 660.3 and 459.9 and 685.3 nm, respectively, and the maximum absorption peak of the **NFBDT** molecule is red-shifted. The maximum absorption peaks of **ITIC** and **NFBDT** are mainly obtained from the S_0_→S_1_ transition, corresponding to the orbital transition of HOMO→LUMO. Therefore, the absorption spectra of **ITIC** and **NFBDT** are substantially identical in terms of the peak position.

### 3.3. The Charge Separation and Recombination at the D/A Interface

After the active layer materials absorb the incident photons, it is necessary for the excitons to undergo diffusion towards the D/A interfaces and be dissociated into CT states prior to their decay into the ground state (S_0_). Then, the CT states are simultaneously dissociated into free electrons and hole carriers, which migrate along the A and D domains and are extracted by the cathode and anode, respectively. However, the charge transfer process accompanied by charge recombination, and the competition between the two processes, will result in very different PCEs of OSCs. It was reported that the donor and acceptor arrangement could be face-on, edge-on and slipped in donor/acceptor interfaces, and the face-on interfaces make the largest contribution to the charge transfer [46,48,49]. In this work, the interface models extracted from the final equilibrium system with a face-on style are mainly considered, which normally have good intermolecular π-π stacking (shown in Figure S6). And the strength of the competition is measured by calculating the rates of the interfacial charge transfer and recombination of the dimer to describe the interfacial exciton dissociation process in detail.

#### 3.3.1. The Frenkel Exciton States and CT States

**Figure 3:**
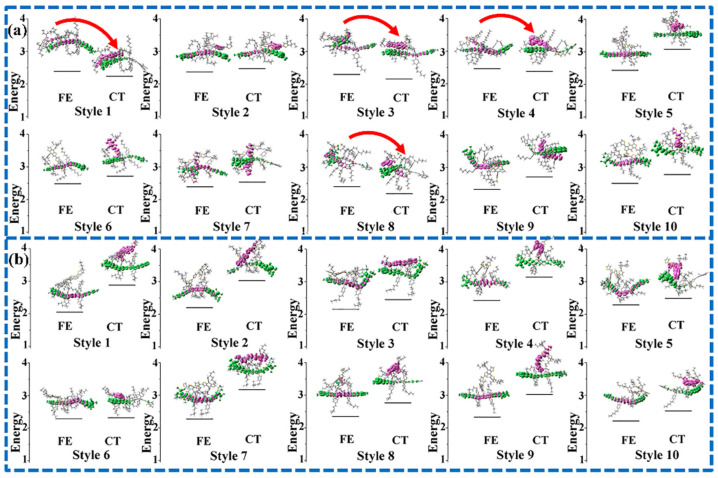
The excitation energies (eV) of FE and CT states of ten dimers extracted from the final equilibrium system for **PBDB-T**/**ITIC** (**a**) and **PBDB-T**/**NFBDT** (**b**) were calculated at the level of CAM-B3LYP/6-31G (d, p).

**Figure 4:**
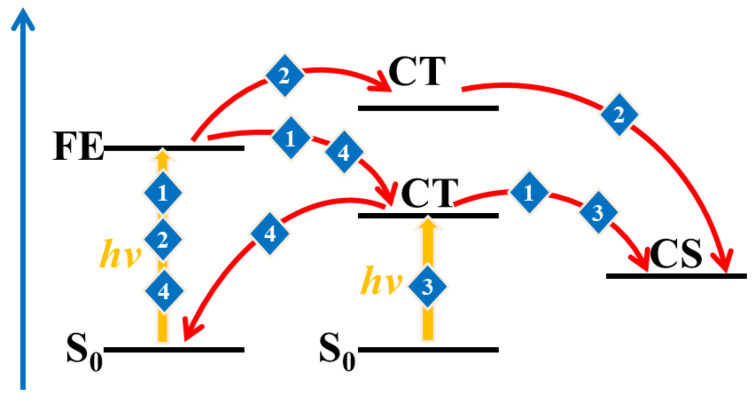
Possible path diagram for describing the photogenerated charge process (1, 2 and 3 are charge separation paths and 4 is charge recombination path, respectively).

The excited-state properties of the D/A interface including the FE states and CT states is a major factor used to evaluate the D/A separation ability. Generally, the excited states with the largest oscillation strength of all electrons and holes on the D or A are called the FE states (the donor materials in the system are the same; only the FE states of acceptor molecules are considered below). The holes are distributed on the donor, and the electrons on the acceptor are called the CT state. The energy of the lowest CT state (CT_1_) and the FE are depicted in Figure 3. In addition, the charge separation paths are also summarized in Figure 4, including the separation process paths and recombination process paths. When the energy of the FE state exceeds that of the CT state, path 1 is preferred; path 2 may be taken for the higher CT state, and path 3 may be taken if the CT state has a higher oscillator intensity. The electrons and holes may also undergo recombination back to the ground state, namely path 4. Relative to **PBDB-T/NFBDT**, **PBDB-T/ITIC** has a lower energy difference between the FE and CT states and more patterns that the energy of the Frenkel exciton state is higher than that of the CT state (such as style 1, style 3, style 4 and style 8, which are shown in Figure 3a). The results indicate that **PBDB-T/ITIC** is favorable for the hot exciton mechanism (path 1), while **PBDB-T/NFBDT** without a higher energy of the FE state than the CT state implies that the photogenerated charge process may be carried out through the IEF mechanism (path 2) or direct excitation (path 3), which may be relatively difficult for the charge separation process. When the FE state has a CT fraction, and the CT state has an FE fraction for **PBDB-T/ITIC**, it is defined as the hybrid FE/CT state. The excited state of **PBDB-T/ITIC** has obvious characteristics of a hybrid FE and CT state. The FE/CT state is related to the strong vibration coupling of the system, which can induce the ultrafast CS process and reduce the non-radiated voltage loss [50]. Importantly, due to the incorporation of a degree of FE fraction, it is possible for **PBDB-T/ITIC** to achieve the direct generation of a CT state via light excitation, carrying out the rapid CS process. Compared to **PBDB-T/ITIC**, **PBDB-T/NFBDT** has only one FE/CT state among ten dimers with good π-π stacking. Obviously, **PBDB-T/ITIC** has obvious FE states with higher energies than the CT state, and a more hybrid FE/CT state, which may be one of the reasons for the higher device performance.

#### 3.3.2. Charge Separation and Recombination Rate

The charge separation or recombination rate will be influenced by the Gibbs free energy difference and *λ* from the Marcus theory. *λ* is affected by the change in the geometric structure of the materials and is mainly related to the polarization of the surrounding medium. The values of external reorganization energy only have a little difference due to the same donor and the small geometric change in the acceptor in two systems. Furthermore, both the CS (*λ*_CS_) and CR (*λ*_CR_) processes of the **PBDB-T/ITIC** and **PBDB-T/NFBDT** interfaces were calculated and are presented in Table 1. The reorganization energy of **PBDB-T/NFBDT** is higher than **PBDB-T/ITIC** in both the charge separation and recombination processes. The difference is mainly from the internal reorganization energy, indicating more geometric relaxation of **NFBDT** during the charge transfer process.

The ∆*G*_CS_ and ∆*G*_CR_ are lower than zero, which is consistent with the exothermic reaction in the charge transfer and recombination processes [51]. **PBDB-T/ITIC** has a larger absolute value of ∆*G*_CR_ and a smaller absolute value of ∆*G*_CS_, as shown in Table 1, which is related with the higher LUMO energy level of the **ITIC** molecules. In addition, when the sum of the Gibbs free energy difference and the total reorganization energy is equal to zero, the rate value of the system is the largest. The value of ∆*G* + *λ* is less than 0, namely the absolute value of ∆*G* is higher than *λ*, and the rate, k, of the system increases with the increased ∆*G*. It can be seen that the ∆*G* + *λ* value of both the CS (−0.383 and −0.368 eV, respectively) and CR (−1.18 and −1.14 eV, respectively) are less than 0 for **PBDB-T/ITIC** and **PBDB-T/NFBDT**. The ∆G of **PBDB-T/ITIC** is −0.954, which is larger than **PBDB-T/NFBDT** (−0.967 eV), indicating a larger *k*_CS_ of **PBDB-T/ITIC** in the CS process. During the CR process, **PBDB-T/ITIC** has a lower ∆*G* (−1.701 eV) than **PBDB-T/NFBDT** (−1.687 eV), and may have a smaller *k*_CR_. These results mean that **PBDB-T/ITIC** may have a higher charge separation rate and a lower charge recombination rate. Electron coupling plays a crucial role in determining the final rate, and an effective charge dissociation requires a large *V*_CS_ and a small *V*_CR_. The electronic coupling values (*V*_CS_ and *V*_CR_) of the extracted interface models are calculated (Table S2). Most of the extracted **PBDB-T/ITIC** models have larger *V*_CS_ values than **PBDB-T/NFBDT**, and the remaining have a smaller difference. A larger *V*_CS_ value can be found in **PBDB-T/ITIC**.

**Figure 5:**
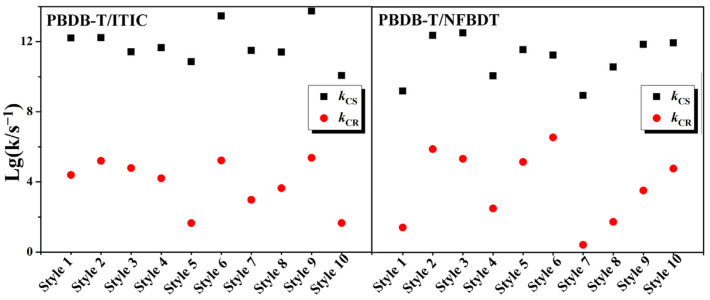
The distribution of charge separation rate *k*_CS_ (s^−1^) and charge recombination rate *k*_CR_ (s^−1^) for **PBDB-T/ITIC** and **PBDB-T/NFBDT** systems.

Based on the Marcus formula, there is a strong correlation between the rate and the square of the electronic coupling. The scatter plot with the *k*_CS_ and *k*_CR_ values of the extracted models as the logarithm of the vertical coordinate and the horizontal coordinate as the name of the dimer are shown in Figure 5. Therefore, the *k*_CS_ and *k*_CR_ values of **PBDB-T/ITIC** and **PBDB-T/NFBDT** decrease sequentially, and the corresponding *V*_CS_ and *V*_CR_ values decrease in the same trend, respectively. Obviously, the *k*_CS_ values of the **PBDB-T/ITIC** systems are higher than those of the **PBDB-T/NFBDT** systems. On the contrary, the distribution of *k*_CR_ indicates that the values of the **PBDB-T/ITIC** systems are lower than those of **PBDB-T/NFBDT**. Compared to **PBDB-T/NFBDT**, the difference between the *k*_CS_ and *k*_CR_ values of **PBDB-T/ITIC** is larger, which means a larger charge separation and a smaller charge recombination rate, facilitating effective charge separation. The higher *k*_CS_ and lower *k*_CR_ of **PBDB-T/ITIC** demonstrates a greater charge separation efficiency than **PBDB-T/NFBDT**.

## 4. Conclusions

In summary, the geometric structure, absorption spectrum, *V*_OC_, ∆*E* and interface parameters were concluded and analyzed using DFT methods to find the reason for the performance difference in the two isomers. The geometric structure, absorption spectrum and *V*_OC_ of the two molecules are similar and have sufficient ∆*E* values for the dissociation of exciton. However, the lowest excited state of **ITIC** has a larger ∆*q*, which may have a positive impact on the charge separation process. By analyzing the main excited states at the interfaces, it was found that the **PBDB-T/ITIC** interface has a more matching relative position between the FE and CT states, which is conducive to having more separation paths and increasing the efficiency of charge separation. Furthermore, there are more FE/CT states in the **PBDB-T/ITIC** systems, indicating a rapid charge separation process. Compared with **PBDB-T/NFBDT**, **PBDB-T/ITIC** presented a larger *k*_CS_ and a smaller *k*_CR_. The results showed that more FE/CT hybridization states, more charge separation paths and the excellent interface parameters of **PBDB-T/ITIC** may be the main reasons for its excellent performance. This work contributes to the study of structural differences in the performance of acceptors, and also provides an idea for the design of new materials for OSCs.

## Figures and Tables

**Table 1:** The internal reorganization energy (*λ_i-CS_* (eV) and *λ_i-CR_* (eV)), external reorganization energy (*λ*_s_ (eV)), total reorganization energy (*λ*_CS_ (eV) and *λ*_CR_ (eV)) and Gibbs free energy difference (Δ*G*_CS_ (eV) and Δ*G*_CR_ (eV)) for the **PBDB-T/ITIC** and **PBDB-T/NFBDT** interfacial models.

	*λ_i-CS_*	*λ_i-CR_*	*λ_s_*	*λ_CS_*	*λ_CR_*	∆*G_CS_*	∆*G_CR_*
**PBDB-T/ITIC**	0.241	0.191	0.330	0.571	0.521	−0.954	−1.701
**PBDB-T/NFBDT**	0.270	0.220	0.329	0.599	0.549	−0.967	−1.687

## Data Availability

Not applicable.

## Supplementary Materials

The following supporting information can be downloaded at https://www.mdpi.com/article/10.3390/molecules28196968/s1, Computational details, Figure S1: Time–potential curves of PBDB-T/ITIC and PBDB-T/NFBDT in NVT process; Figure S2: Time–potential curves of PBDB-T/ITIC and PBDB-T/NFBDT in NPT process; Figure S3: (a) PBDB-T/ITIC and (b) PBDB-T/NFBDT system via MD simulation; Figure S4: Absorption spectra of ITIC and NFBDT calculated at the PBE0/6-31G(d, p) level; Figure S5: The optimized geometric structures of the side and half side of ITIC and NFBDT molecules, with red as the plane; Figure S6. Typical interface models (the gray and red represent PBDB-T and ITIC/NFBDT, respectively) in PBDB-T/ITIC (a) and PBDB-T/NFBDT (b) systems extracted from MD; Table S1: Computed orbital energy (*E*_HOMO_ and *E*_LUMO_), energy driving force (∆*E*_LUMO_ and ∆*E*_HOMO_), open circuit voltage (*V*_OC_) and transferred charge number (∆*q*) of ITIC and NFDFT; Table S2: Electronic coupling values of charge separation *V*_CS_ (eV) and charge recombination *V*_CR_ (eV) of PBDB-T/ITIC and PBDB-T/NFBDT.
