# Supplementary material for: A Theoretical Study on the Underlying Factors of the Difference in Performance of Organic Solar Cells Based on ITIC and Its Isomers
Source: Molecules. 2023 Oct 7;28(19):6968. doi: 10.3390/molecules28196968 (PMC10574239; doi:10.3390/molecules28196968)
Supplement: Supplementary file 1 [file molecules-28-06968-s001.zip › molecules-2588482-supplementary.pdf]

Supporting Information

A theoretical study on the underlying factors for the difference in performance of organic solar cells based on ITIC and its isomer

Si-Qi Huang [1], Li-Li Wang [2,3], Qing-Qing Pan [3], Zhi-Wen Zhao [1,*], Ying Gao [2,*] and Zhong-Min Su [3,4,]

1 College of Chemical Engineering, Hubei University of Arts and Science, Xiangyang 441053, China; huangsiqi61@outlook.com
2 Jilin Provincial Key Laboratory of Straw–Based Functional Materials, Institute for Interdisciplinary Biomass Functional Materials Studies, Jilin Engineering Normal University, Changchun 130052, China; 18523778447@163.com
3 School of Chemistry and Environmental Engineering, Changchun University of Science and Technology, Jilin Provincial Science and Technology Innovation Center of Optical Materials and Chemistry, Jilin Provincial International Joint Research Center of Photo-Functional Materials and Chemistry, Changchun 130022, China; panqq349@nenu.edu.cn (Q.-Q.P.); zmsu@nenu.edu.cn (Z.-M.S.)
4 State Key Laboratory of Supramolecular Structure and Materials, Institute of Theoretical Chemistry, College of Chemistry, Jilin University, Changchun 130021, China
* Correspondence: zhaozw@hbuas.edu.cn (Z.-W.Z.); gaoy029@163.com (Y.G.)

Number of pages: S4

Number of figures: 6

Number of tables: 2

The electronic coupling V_{DA} have been evaluated by the generalized Milliken-Hush (GMH) formalism [1] which refers to a vertical transition from the initial state to the final state. V_{DA} is written as

$$V_{DA} = \frac{\mu_{tr}\Delta E}{\sqrt{(\Delta\mu)^2 + 4(\mu_{tr})^2}} \quad \text{(S1)}$$

Where $\Delta\mu$ represents the dipole moment difference between the initial and final states, ΔE is the energy difference and μ_{tr} is the transition dipole between these two states.

Generally, for exciton, the electron and the hole often experience a strong attraction, which is called exciton binding energy (E_b) [2]. The E_b has to be overcome for the charges to escape from the D/A interface and migrate towards the cathode and the anode.

$$E_b = \Delta E_{H-L} - E_{S1} \quad \text{(S2)}$$

ΔE_{H-L} is the energy difference between HOMO and LUMO, and E_{S1} is the first singlet excitation energy of acceptor. Based on this formulation, we calculated E_b at the B3LYP/6-31G(d, p) level. Gibbs free energy change (ΔG_{CR}) of charge recombination process can be estimated with[3]

$$\Delta G_{CR} = E_{IP}(D) - E_{EA}(A) \tag{S3}$$

Where E_{IP}(D) represents the ionization potential of the donor, E_{EA}(A) is the electron affinity of the acceptor. As an approximation, the Gibbs free energy change (ΔG_{CS}) of charge-separation process is estimated from the Rehm-Weller equation [3].

$$\Delta G_{CS} = -\Delta G_{CR} - \Delta E_{S1} - \Delta E_b \tag{S4}$$

E_{S1} and E_b are the energy of lowest excited state of free-base donor and exciton binding energy, respectively.

The reorganization energy λ is normally decomposed into internal energy (λ_i) and external energy (λ_s). The internal reorganization energy can be estimated from the exciton dissociation and charge recombination processes[4]. The reorganization energy of the charge dissociation, $\lambda_{i\text{-}CS}$, can be estimated according to the eqs. (S5-S7):

$$\lambda_{i1} = \left[E^{D^*}(Q_P) + E^A(Q_P)\right] - \left[E^{D^*}(Q_R) + E^A(Q_R)\right] \tag{S5}$$

$$\lambda_{i2} = \left[E^{D^+}(Q_R) + E^{A^-}(Q_R)\right] - \left[E^{D^+}(Q_P) + E^{A^-}(Q_P)\right] \tag{S6}$$

$$\lambda_{i\text{-}CS} = (\lambda_{i1} + \lambda_{i2})/2 \tag{S7}$$

The reorganization energy of the charge recombination process, $\lambda_{i\text{-}CR}$, is given by:

$$\lambda_{i2} = \left[E^{D^+}(Q_R) + E^{A^-}(Q_R)\right] - \left[E^{D^+}(Q_P) + E^{A^-}(Q_P)\right] \tag{S8}$$

$$\lambda_{i3} = \left[E^{D}(Q_R) + E^{A}(Q_R)\right] - \left[E^{D}(Q_P) + E^{A}(Q_P)\right] \tag{S9}$$

$$\lambda_{i\text{-}CR} = (\lambda_{i2} + \lambda_{i3})/2 \tag{S10}$$

Where λ_{i1} represents the difference between the energy of the excited-state (D*A) reactants in the geometry characteristic of the products and that in their equilibrium geometry, λ_{i2} is the difference between the energy of the ionic-state (D^+A^-) reactants in the geometry characteristic of the reactants and that in their equilibrium geometry, λ_{i3} is the difference between the energy of the ground-state (DA) reactants in the characteristic of the products and that in their equilibrium geometry. Q_P and Q_R are the equilibrium geometries of the products and reactants, respectively.

In the interface model, external reorganization energy accounts for an important fraction of λ, and can't been ignored. The calculation of external reorganization energy λ_s is based on the classical dielectric continuum model with the quantum mechanics methods and it is given by

$$\lambda_s = \Delta q^2 \left(\frac{1}{2d_D} + \frac{1}{2d_D} - \frac{1}{d_{DA}}\right)\left(\frac{1}{\epsilon_{op}} - \frac{1}{\epsilon_o}\right) \tag{S12}$$

Where d_{DA} represents the mass-center distance between the donor and the acceptor, d_D and d_A are the radii of the donor and acceptor, respectively. ϵ_{op} is the optical-frequency dielectric constant and the typical value (2.25) was used in our calculations. ϵ_o is the zero-frequency dielectric constant of the medium.

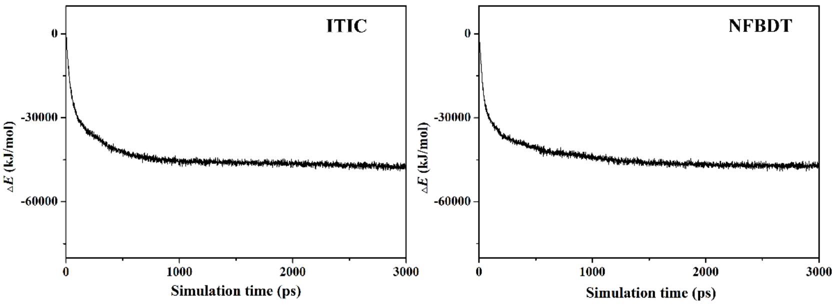

Figure S1. Time-potential curves of **PBDB-T/ITIC** and **PBDB-T/NFBDT** in NVT process.

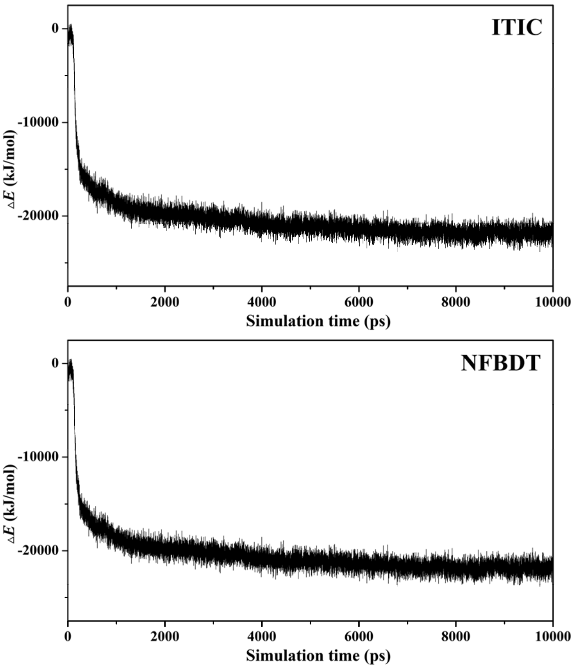

Figure S2. Time-potential curves of **PBDB-T/ITIC** and **PBDB-T/NFBDT** in NPT process.

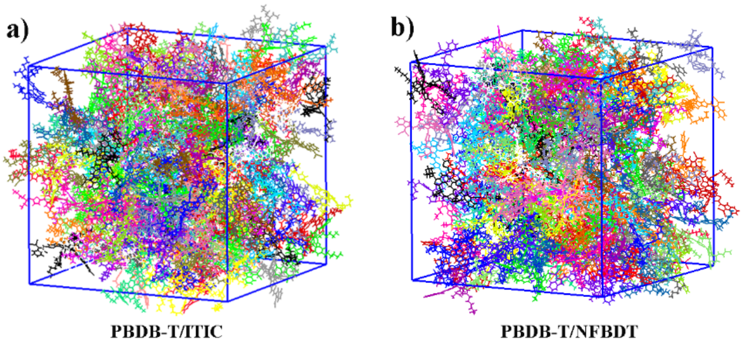

Figure S3. (a) **PBDB-T/ITIC** and (b) **PBDB-T/NFBDT** system by MD simulation.

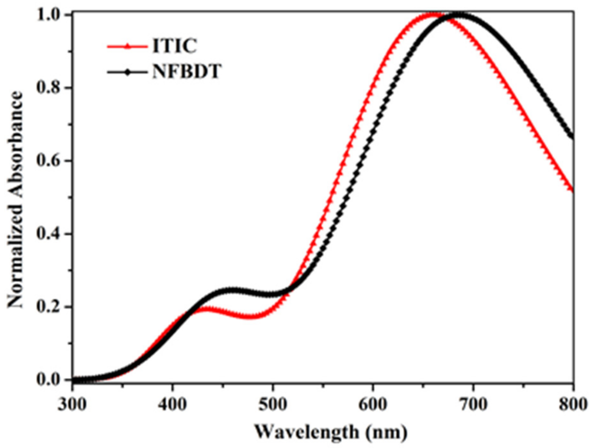

Figure S4. Absorption spectra of **ITIC** and **NFBDT** calculated at the PBE0/6-31G(d, p) level.

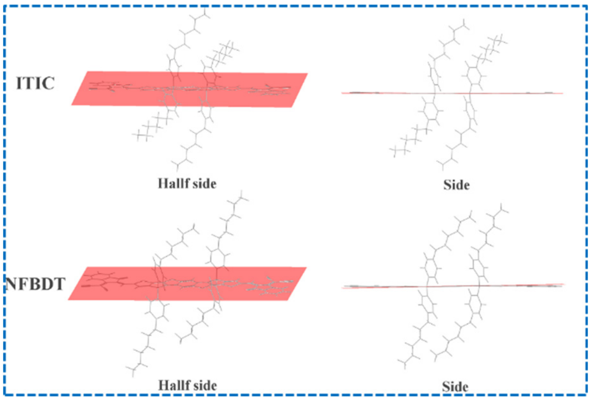

Figure S5. The optimized geometric structures of the side and half side of **ITIC** and **NFBDT** molecules, with red as the plane.

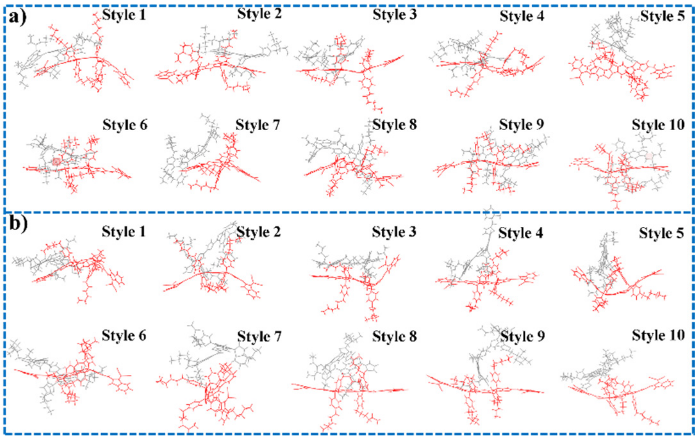

Figure S6. Typical interface models (the gray, red represent **PBDB-T**, **ITIC/NFBDT**, respectively) in **PBDB-T/ITIC** (a) and **PBDB-T/NFBDT** (b) systems extracted from MD.

Table S1. Computed orbital energy (E_{HOMO} and E_{LUMO}), energy driving force (ΔE_{LUMO} and ΔE_{HOMO}), open circuit voltage (V_{OC}) and transferred charge number (Δq) of **ITIC** and **NFDFT**.

	E_{HOMO}	E_{LUMO}	ΔE_{LUMO}	ΔE_{HOMO}	V_{OC}
**ITIC**	-5.44	-3.33	1.05	0.41	1.40
**NFBDT**	-5.39	-3.35	1.03	0.36	1.38
**PBDB-T**	-5.03	-2.38	—	—	—

Table S2. Electronic coupling values of charge separation V_{CS} (eV) and charge recombination V_{CR} (eV) of **PBDB-T/ITIC** and **PBDB-T/NFBDT**.

	**PBDB-T/ITIC**		**PBDB-T/NFBDT**	
	V_{CS}	V_{CR}	V_{CS}	V_{CR}
1	0.0294	0.4298	0.0008	0.0030
2	0.0298	1.0807	0.0311	0.5341
3	0.0119	0.6757	0.0367	0.2824
4	0.0156	0.3458	0.0022	0.0109
5	0.0062	0.0182	0.0120	0.2298
6	0.1255	1.1080	0.0085	1.1490
7	0.0130	0.0833	0.0006	0.0010
8	0.0117	0.1798	0.0039	0.0045
9	0.1718	1.3111	0.0173	0.0352
10	0.0024	0.0183	0.0191	0.1487

Molecular coordinates:

ITIC

C	-1.7132582011	0.4329413535	5.2143761865
C	-1.8713850438	0.8245501494	6.5703561171
C	-4.0063675825	0.3644509512	5.7482734584
C	-3.2864473502	0.7817377091	6.8541674183
C	-7.0609488809	0.6803967552	7.6291005395
C	-5.6995392511	0.7180193923	7.3365580285

C	-5.392656249	0.3300392185	6.0147369315
S	-6.8219874816	-0.0882830693	5.1186505277
H	-7.5058064308	0.9410975763	8.5825953521
S	-4.259889452	1.1434796699	8.2572380933
C	-3.0773801547	0.1128318143	4.5592244573
C	0.6488570611	0.6872532646	5.4588124
C	0.4903973539	1.0838791765	6.8133054296
C	-7.8400313756	0.2608391306	6.5382028947
C	2.7843980483	1.142698548	6.2822228272
C	2.0646761153	0.7219702507	5.1774949385
C	5.8420932063	0.7905114694	4.4130059957
C	4.4795560541	0.7653908787	4.7015806098
C	4.1716195967	1.1655173294	6.0195534896
S	5.6014307852	1.5792101378	6.9171791282
H	6.2878852458	0.5193503984	3.462865342
S	3.0395642023	0.3432672491	3.7799045409
C	1.8537997035	1.4140334427	7.4654369934
C	6.6210053411	1.2121837618	5.503201162
C	-0.7732066815	1.1571797321	7.3769415058
H	-0.9120231395	1.4647082488	8.4083840204
C	-0.4495939444	0.3569504325	4.6514097556
H	-0.3111481965	0.0482835597	3.6201904758
C	11.2152613453	2.1184393008	7.105231422
C	10.2762151434	2.3709275968	8.1219433116
C	10.4936882137	1.6597781639	5.8879321986
C	9.0621926099	1.6460012679	6.2111889305
C	8.9137335072	2.0908110769	7.6169249729
O	7.8749344753	2.2111330608	8.2564421997
C	11.1065617306	1.3192911562	4.6943687649
C	12.5205878045	1.3695431208	4.4924339972
C	10.4063986221	0.879196845	3.5287546155
N	13.6664191931	1.3951132596	4.2827648319
N	9.8681857493	0.5174997321	2.560160801
C	12.5746397951	2.3212887396	7.3717637577
H	13.3388258669	2.1431721695	6.6281313867
C	12.946708314	2.7687791178	8.6432027902
H	13.9996708107	2.9276926701	8.8548724989
C	11.998537901	3.0151955699	9.643433664
H	12.3231185199	3.3623462837	10.6195709241
C	10.6414655247	2.8151553388	9.385208029
H	9.8783567555	2.9960648521	10.1353716698
C	8.0327262736	1.2867535407	5.3676092058
H	8.3271934789	0.9881443226	4.3679358889
C	-12.4318948352	-0.6679715015	4.9423061622

C	-11.4939155919	-0.9051222212	3.9209270412
C	-11.7103034609	-0.2134851988	6.1611609856
C	-10.2798901081	-0.186248471	5.8339851486
C	-10.132153681	-0.6187634538	4.4243283019
O	-9.094324952	-0.7262593366	3.7809635548
C	-12.3223104326	0.1128987891	7.3591048962
C	-13.7353410808	0.0504392855	7.5645646744
C	-11.6221476522	0.5490856708	8.5261858966
N	-14.8803554339	0.0146736258	7.7771854096
N	-11.0838905595	0.9072486628	9.4960677614
C	-13.7904467502	-0.8790994724	4.678002611
H	-14.5538309744	-0.7128531591	5.4252016473
C	-14.1627704333	-1.319179574	3.4040530181
H	-15.2150989283	-1.4844534833	3.1941075276
C	-13.2156623342	-1.5502278203	2.399155305
H	-13.5404198761	-1.8919264304	1.4211561608
C	-11.859414531	-1.3419176611	2.6551466756
H	-11.0971205657	-1.5108809226	1.9013799384
C	-9.250733152	0.1743992476	6.6773598076
H	-9.5445577002	0.4631458412	7.6801192578
C	-3.455918726	1.0996543113	3.4317753541
C	-4.4823989255	0.7848646345	2.5329850328
C	-2.867802967	2.3678759352	3.3310454316
C	-4.8979711884	1.6970052282	1.5615648536
H	-4.9608562486	-0.1882420345	2.5769747201
C	-3.2868322419	3.2740244779	2.3605706857
H	-2.0774731483	2.6559075068	4.0157156042
C	-4.3068666384	2.9602724559	1.4512354252
H	-5.6982214003	1.4075424686	0.8888861059
H	-2.8089319389	4.2497379851	2.3084982417
C	-3.0611292967	-1.3572246753	4.0920233727
C	-2.5371017326	-1.6999340908	2.8366315452
C	-3.48086635	-2.395264645	4.9317244102
C	-2.4372749847	-3.0319424908	2.4416419296
H	-2.2240006898	-0.918061923	2.1521855229
C	-3.3807725266	-3.7273090761	4.5299941481
H	-3.8933097263	-2.167314989	5.9090064014
C	-2.8552279753	-4.073700536	3.2804922262
H	-2.0345159416	-3.265754832	1.4589639113
H	-3.7247357492	-4.5106959381	5.2009623359
C	2.2307848121	0.452497428	8.6142495757
C	3.2473970356	0.7926975838	9.5147168925
C	1.6467359924	-0.815570084	8.7389805871
C	3.6602134539	-0.0962682477	10.5086146599

H	3.7184574578	1.7685773055	9.4554324784
C	2.0597359839	-1.6967742556	9.7346844455
H	0.8589227647	-1.1195905707	8.0582970523
C	3.0750461955	-1.3601287103	10.6413213014
H	4.4496725889	0.2139933356	11.1847479366
H	1.5798487925	-2.6699667391	9.8102175811
C	1.8344021462	2.8940808502	7.9022650672
C	1.286588308	3.2629140376	9.1395414634
C	2.2747684814	3.9145912703	7.0511594952
C	1.1787777261	4.6032242346	9.50473103
H	0.9589484243	2.4956940849	9.8337560518
C	2.169052332	5.2541831428	7.4238873806
H	2.7078255441	3.666695601	6.0878500217
C	1.6146852542	5.6269789087	8.6537057356
H	0.7542743558	4.8573579534	10.4730454145
H	2.5298910748	6.022988841	6.7448388588
C	1.4528461136	7.0835993769	9.0283180307
H	1.4796657519	7.1895587551	10.1199391526
H	2.3011322556	7.6604718841	8.638651729
C	0.1424666222	7.6958016166	8.4935401526
H	0.1125676065	7.5750856977	7.4023424457
H	-0.7070087423	7.120070804	8.8846469364
C	-0.0284927137	9.1764722893	8.8520245004
H	0.8305920766	9.7451043858	8.4681018275
H	-0.0036988695	9.2924197503	9.9450014011
C	-1.3251245012	9.7864074075	8.3056984234
H	-2.1842062041	9.2178997704	8.6896861409
H	-1.3490783629	9.66744336	7.2129467539
C	-1.5035279975	11.2689141972	8.6555511201
H	-0.6440236632	11.8364574832	8.2735388987
H	-1.4833265415	11.3881431865	9.7474227723
C	-2.7988867656	11.8684270804	8.1003564322
H	-3.6778751197	11.3429322914	8.4905677594
H	-2.8981241302	12.9254201061	8.3676696824
H	-2.8316531378	11.7975778018	7.0071999191
C	-4.7057248115	3.977541141	0.3959900886
H	-4.7580534918	4.9655419454	0.8732618818
H	-3.8918555626	4.0565373594	-0.3400136468
C	-6.0194346827	3.7162517086	-0.349626498
H	-5.9538388284	2.7676696749	-0.8988094413
H	-6.8357451666	3.5961857584	0.3750493848
C	-6.3722852218	4.8372624298	-1.3353054331
H	-6.4491221829	5.7890813521	-0.790690397
H	-5.5476883589	4.9644511104	-2.0513070715

C	-7.6754392618	4.5870376355	-2.1034513356
H	-8.5001873947	4.4583976368	-1.3879711024
H	-7.5985055848	3.6359308936	-2.6496864722
C	-8.0316002964	5.7074300643	-3.0883275319
H	-8.1097914222	6.6572492402	-2.5420160603
H	-7.2069221968	5.8364271865	-3.8024816437
C	-9.3334510409	5.4493785401	-3.8526081921
H	-10.1822878852	5.3490354547	-3.1666399237
H	-9.5607627922	6.2665349022	-4.5448006081
H	-9.2726243478	4.5250009225	-4.4382359146
C	-2.7057537922	-5.5203260907	2.8643988265
H	-3.4686945706	-6.1276483936	3.3669612239
H	-2.8925506114	-5.6155576852	1.7871081682
C	-1.3116603722	-6.096073716	3.1844813847
H	-0.5489569863	-5.4875113147	2.6803869487
H	-1.1197796061	-5.9902796883	4.2605473476
C	-1.1521384415	-7.5649627992	2.7748453842
H	-1.9135515683	-8.1699681122	3.2876182899
H	-1.3580705519	-7.6679735969	1.6998070269
C	0.2387371435	-8.1327959126	3.0815320339
H	0.9988611027	-7.5315453554	2.5624557446
H	0.4478735195	-8.0213310152	4.1551239676
C	0.4033749061	-9.605567698	2.685809543
H	-0.3536756855	-10.2067073875	3.2076109548
H	0.1920471947	-9.7177460759	1.6136910661
C	1.7971921947	-10.1609910646	2.992672756
H	1.8819396574	-11.2135400402	2.7037258755
H	2.5729039352	-9.6049861768	2.4540121327
H	2.0248458525	-10.0903967355	4.0624116985
C	3.4983248719	-2.3670966888	11.6972153787
H	2.5928260576	-2.8065253707	12.1375443713
H	4.0099364843	-3.2026107461	11.1971441325
C	4.3960163732	-1.8427473786	12.8234242143
H	5.3397475244	-1.4709266185	12.4032671688
H	3.913934246	-0.9819779893	13.3063290002
C	4.7086297304	-2.9097169067	13.8802433984
H	5.1839434379	-3.774020071	13.3947959885
H	3.7679808692	-3.2831572643	14.3093622422
C	5.6139522647	-2.4012505309	15.0082692275
H	6.5543121889	-2.0270127767	14.5788646458
H	5.1386235066	-1.5367749315	15.4936981892
C	5.9312163757	-3.4636094347	16.0680958085
H	6.40811081	-4.3263404407	15.5833069564
H	4.9918723473	-3.8385776221	16.4967371705

C	6.834390488	-2.9458502236	17.1912421491
H	7.0452329346	-3.7274014515	17.928303307
H	7.7942777298	-2.5930907434	16.7971733464
H	6.3678678339	-2.1072463593	17.7206084304

NFBDT:

C	1.0166628555	-0.9321167151	0.0463367112
C	1.3338163004	0.4685180678	0.0372174668
C	3.459133827	-0.5105261394	0.1159864499
C	2.7479198821	0.6729673483	0.0710556814
C	6.3824795641	1.5058547161	0.2793923217
C	5.0386452703	1.1569909676	0.2117388794
C	4.8602268218	-0.2294843567	0.1882648156
S	6.328244405	-1.1264079559	0.2530581592
H	6.7687147557	2.5184774691	0.3043548241
C	-1.3337474118	-0.4676820254	-0.0371865324
C	-1.016588863	0.9329526504	-0.0463844267
C	7.252305519	0.3962970015	0.3163517754
C	-3.4590530145	0.511372222	-0.1160646937
C	-2.7478537827	-0.6721258155	-0.0709859333
C	-6.3824390013	-1.504966954	-0.2791772392
C	-5.0385970692	-1.1561245472	-0.2115178202
C	-4.8601498824	0.2303502594	-0.1883011564
S	-6.3281381857	1.1272990747	-0.2534166033
H	-6.7687039647	-2.5175830367	-0.3039383922
C	-7.2522337152	-0.3953955208	-0.3164383331
C	0.2846259969	1.4048873812	-0.0111459439
H	0.4994667117	2.4666411794	-0.0134613807
C	-0.284553114	-1.4040509685	0.0111344367
H	-0.4993928322	-2.4658061216	0.0134114949
C	-11.952761447	1.1231833536	-0.5780405937
C	-11.0756286388	2.2221689373	-0.5295134927
C	-11.1538088289	-0.1311452434	-0.5258749513
C	-9.7419273554	0.2572161831	-0.4474958694
C	-9.6807182382	1.7356846397	-0.4452463694
O	-8.6784667329	2.4406931567	-0.3870013859
C	-11.6929002128	-1.4061725852	-0.548239246
C	-13.0958344696	-1.667010444	-0.6272411896
C	-10.9184695387	-2.6059692133	-0.4945571975
N	-14.2299273733	-1.9266541845	-0.6910219023
N	-10.3185756573	-3.6045289347	-0.4525601003
C	-13.3301350974	1.3579827379	-0.6620054943
H	-14.049042037	0.5515772205	-0.702251862
C	-13.7817165389	2.6812262297	-0.6949032783

H	-14.8491470219	2.8685347721	-0.7604883604
C	-12.8945868191	3.7629978361	-0.6455341666
H	-13.2800293444	4.7775773346	-0.6730409786
C	-11.5195673459	3.5366864766	-0.5613194878
H	-10.8023090449	4.3501687331	-0.5210492207
C	-8.6540561292	-0.5904548785	-0.3924987973
H	-8.8810280987	-1.6505166308	-0.4101626279
C	11.9529217931	-1.1222917473	0.5761907222
C	11.0758009625	-2.2212700208	0.5272761355
C	11.1539079842	0.1320500697	0.5252628981
C	9.7420195613	-0.2563022474	0.4469535079
C	9.680846402	-1.7347702385	0.4438382746
O	8.6785849826	-2.4397676983	0.3856218838
C	11.6929471133	1.4070812747	0.5486791416
C	13.0958922081	1.6679152882	0.6275158565
C	10.9184410112	2.6068924054	0.4964335518
N	14.2299892905	1.9275658579	0.6911958748
N	10.3184815247	3.605461838	0.4556187767
C	13.3303403485	-1.3571091951	0.6593649701
H	14.0492403819	-0.5507094199	0.6998344679
C	13.7819774985	-2.6803614518	0.691119806
H	14.8494444792	-2.8676829451	0.7560694372
C	12.8948567509	-3.7621249367	0.6414104952
H	13.2803414638	-4.7767120338	0.6680313182
C	11.5197923979	-3.5357958562	0.5579799966
H	10.8025402075	-4.3492710974	0.5174589193
C	8.6541266455	0.5913678281	0.3923769379
H	8.8810768187	1.6514292243	0.4103546935
S	2.4699464308	-1.9465657339	0.1082334103
S	-2.4698604111	1.9474033931	-0.1084390681
C	3.6889223753	1.889439033	0.1269080422
C	-3.688890336	-1.8885873884	-0.1265165389
C	-3.3729210096	-2.7074493627	-1.4013647268
C	-2.5698060939	-3.854248284	-1.354085876
C	-3.8199313853	-2.2725914306	-2.6561323912
C	-2.234280863	-4.5430064485	-2.519710515
H	-2.2215238139	-4.2318844565	-0.3986009854
C	-3.4819991597	-2.9645051455	-3.8171817305
H	-4.4449222331	-1.3883183474	-2.7298253787
C	-2.6825447543	-4.1139956685	-3.7741359138
H	-1.6196235456	-5.437191984	-2.4481396205
H	-3.8538424785	-2.6076690276	-4.7745911856
C	-3.6797173917	-2.7438411848	1.1565444615
C	-4.4375888913	-3.9245462236	1.2064643896

C	-2.9857805059	-2.3624443626	2.3092351965
C	-4.4886390151	-4.695718777	2.3637823562
H	-4.9756382761	-4.2573568468	0.3239303619
C	-3.0381181356	-3.1417110475	3.4667326224
H	-2.3928706653	-1.4547138528	2.311608927
C	-3.7866892235	-4.3216371699	3.5186492867
H	-5.0770392019	-5.6099598124	2.3674947622
H	-2.4769442879	-2.8251495283	4.3426399316
C	3.6797447854	2.7450818253	-1.1558813875
C	4.4375027861	3.9258744415	-1.2054127332
C	2.9859196714	2.3639465247	-2.3087289852
C	4.488536682	4.6973950794	-2.362502264
H	4.975471711	4.2584762525	-0.3227506155
C	3.0382393818	3.1435588372	-3.4659914959
H	2.3931060391	1.4561523116	-2.3113950276
C	3.786686296	4.3235848447	-3.5175140548
H	5.0768336489	5.6117037167	-2.3659087365
H	2.4771460455	2.8272027153	-4.342024759
C	3.3728527514	2.7078668804	1.4020188142
C	3.8197427019	2.2725435688	2.6566758355
C	2.5696998225	3.8546428348	1.3550879224
C	3.4815983085	2.9639585786	3.817950847
H	4.4447748124	1.3882784819	2.7300957532
C	2.2339656609	4.5429143729	2.5209486486
H	2.2215239286	4.2326416739	0.3997081566
C	2.6820451984	4.1134021468	3.7752598268
H	3.8533219833	2.606761749	4.7752724761
H	1.6192466069	5.4370787016	2.4496517656
C	3.856537089	5.1543957042	-4.7793561674
H	2.9003942185	5.0878126314	-5.3137320368
H	3.9904819152	6.2107196279	-4.514433877
C	4.991812386	4.730743997	-5.7332958088
H	5.9508389412	4.7939105139	-5.2022898277
H	4.8611732393	3.6734456631	-5.9995527909
C	5.0467177625	5.5827880412	-7.0062663936
H	5.1779746952	6.6387414576	-6.7297067139
H	4.0777529419	5.5246414012	-7.5227389744
C	6.1617177229	5.1714285552	-7.975619215
H	6.0302195549	4.1162935564	-8.2550459121
H	7.1316287734	5.2290527977	-7.4613693556
C	6.211564758	6.0282343403	-9.2464710705
H	6.3445078	7.0820613039	-8.9663468394
H	5.2411847163	5.9717484061	-9.7584072772
C	7.3241056314	5.6128676357	-10.213660169

H	7.1990617295	4.57379386	-10.5390126573
H	7.3323023413	6.2423614973	-11.1093808695
H	8.3100272455	5.6934320469	-9.7419734626
C	-3.8565925848	-5.1520532192	4.7807484386
H	-3.9907317028	-6.2084351266	4.516155176
H	-2.900401253	-5.0854672116	5.3150387457
C	-4.9917245775	-4.727918589	5.734641484
H	-4.8609015828	-3.6705523879	6.0005377598
H	-5.9508067759	-4.7911119437	5.2037394392
C	-5.0466525063	-5.5795317843	7.0078998888
H	-5.1781409005	-6.6355519161	6.7317051165
H	-4.0776196436	-5.5213980845	7.5242467492
C	-6.1614637949	-5.1676371044	7.977241758
H	-6.0297312517	-4.1124334269	8.2562980191
H	-7.1314465452	-5.2252478176	7.4631253824
C	-6.2113279919	-6.0240051271	9.2483886061
H	-5.2408720101	-5.9675445383	9.7601839251
H	-6.3445211911	-7.0778991742	8.9686368398
C	-7.3236622922	-5.6080839857	10.2155769668
H	-7.3318791817	-6.2372757321	11.1115097237
H	-7.1983616686	-4.568927002	10.5405644554
H	-8.3096593024	-5.6886006912	9.7440393435
C	2.2871516047	4.8441917503	5.0393234085
H	3.0630869094	4.7040775309	5.8020782587
H	2.2388873475	5.9222728794	4.8400022803
C	0.9313288328	4.3794975609	5.6080555935
H	0.1573477391	4.5117623143	4.8402198564
H	0.9773675322	3.3008011857	5.8085901884
C	0.5245093018	5.1261066234	6.8837101631
H	0.4940914349	6.2056475926	6.6777390098
H	1.3006009853	4.9868049522	7.6498385627
C	-0.8301473039	4.6794924234	7.4466114793
H	-0.8007714113	3.600054619	7.6532174038
H	-1.6056209262	4.8187758149	6.679806549
C	-1.2413767401	5.4263770903	8.7215054096
H	-0.4684513286	5.2844706387	9.4890971211
H	-1.2678739651	6.5050261029	8.5153136313
C	-2.59774214	4.9784812659	9.2738663652
H	-2.5918087346	3.9110805517	9.5224206174
H	-2.8621297587	5.5292007692	10.1823750514
H	-3.3971222677	5.1413756419	8.5420851691
C	-2.288027135	-4.8454657214	-5.0379200855
H	-2.240289186	-5.9234924635	-4.8381634959
H	-3.0639278678	-4.7052860664	-5.8006967781

C	-0.9320007337	-4.3817138932	-5.6069366937
H	-0.977510749	-3.3030980635	-5.8080190627
H	-0.1580394103	-4.5139804742	-4.8390807807
C	-0.5256573324	-5.1291916859	-6.8822324948
H	-0.4957217804	-6.2086352734	-6.6756801325
H	-1.3017662233	-4.9899426712	-7.6483528813
C	0.8291458073	-4.683526332	-7.44554042
H	0.800251571	-3.6041942753	-7.652764058
H	1.6046407689	-4.8227350155	-6.6787435779
C	1.239866983	-5.4313415558	-8.7200510475
H	0.4669109257	-5.2895246377	-9.4876283381
H	1.2658938263	-6.5098823642	-8.5132357642
C	2.5963687307	-4.984387366	-9.2728426457
H	2.860400348	-5.5357619946	-10.1810576291
H	2.5908844561	-3.917131198	-9.5220270561
H	3.3957620958	-5.1472078853	-8.5410597545

References:

1. A. A. Voityuk, *J. Chem. Phys.*, **2006**, *124*, 064505.
2. T. M. Clarke and J. R. Durrant, *Chem. Rev.*, **2010**, *110*, 6736.
3. G. J. Kavarnos and N. J. Turro, *Chem. Rev.*, **1986**, *86*, 401.
4. V. Lemaur, M. Steel, D. Beljonne, J.-L. Brédas and J. Cornil, *J. Am. Chem. Soc.*, **2005**, *127*, 6077.
